# Supplementary material for: Locomotor activity patterns of takin (Budorcas taxicolor) in a temperate mountain region
Source: PLoS One. 2020 Jul 13;15(7):e0235464. doi: 10.1371/journal.pone.0235464 (PMC7357786; doi:10.1371/journal.pone.0235464)
Supplement: S1 Table — (DOCX) [file pone.0235464.s001.docx]

**S1 Table. Takin-specific GPS collar data including individual ID, age, sex, start and end dates of collar deployment, number of fixes after data filtering and number of data separated only by 2 h from** **1 July 2014 to 30 June 2015.**

| Individual ID | Years estimated age* | Sex | Estimated body mass(kg) | Start date of collaring | Number of fixes after data filtering | Number of data separated only by 2 h |
| --- | --- | --- | --- | --- | --- | --- |
| M1 | 12 | Male | 250 | 25 August 2013 | 4141 | 4013 |
| M2 | 10 | Male | 250 | 6 September 2013 | 3583 | 3039 |
| M3 | 13 | Male | 300 | 22 June 2014 | 3075 | 2348 |
| M4 | 12 | Male | 280 | 26 June 2014 | 3345 | 2700 |
| F1 | 8 | Female | 210 | 18 June 2014 | 3640 | 3111 |
| F2 | 10 | Female | 230 | 22 June 2014 | 3682 | 3178 |
| F3 | 8 | Female | 200 | 22 June 2014 | 3221 | 2494 |
| F4 | 7 | Female | 210 | 27 June 2014 | 3306 | 2611 |
| F5 | 7 | Female | 200 | 17 June 2014 | 3704 | 3211 |
| F6 | 6 | Female | 190 | 15 June 2014 | 4209 | 4051 |

* We estimated takin age based on wear degree of molars.
