# Supplementary material for: Locomotor activity patterns of takin (Budorcas taxicolor) in a temperate mountain region
Source: PLoS One. 2020 Jul 13;15(7):e0235464. doi: 10.1371/journal.pone.0235464 (PMC7357786; doi:10.1371/journal.pone.0235464)
Supplement: Attachment — Submitted filename: Response to Reviewers.docx [file pone.0235464.s003.docx]

Reviewer #1: The manuscript describes the locomotion activity pattern of 10 free-living takins that were tagged with GPS collars. The data were correlated with NDVI data and temperature data measured by the GPS-collar.
In my opinion, the study gives no new insight into activity behavior of herbivores in temperated area; former studies which were carried out by camera traps have shown similar results and the hypotheses here (which are not daring anyway) were -as expected- simply confirmed. In addition, the technique used was not exploited in all its possibilities:
1) The authors used GPS-collars from Lotek company. Lotek collars also have 3D-activity loggers; These are much better suited to continuously and high-resolution record the activity of the animals and sometimes even to distinguish between different behaviors (Brown et al. 2014 in Animal Biotelemetry).

I’m sorry. Because of project capital limitation, the Lotak collars GPS7000 of we used were not fitted out with 3D-activity loggers.

2) The spatial data (positions) measured by the GPS collars were not used in spatial analyses (home range calculation, habitat use).

OK. We had used the spatial data (positions) measured by the GPS collars to analyses home range and habitat use of takin.

Yan WB, Zeng ZG, Gong HS, He XB, Liu XY, Si KC, et al. Habitat use and selection by takin in the Qinling Mountains, China. Wildlife Research. 2016; 43(8):671-80.

Yan WB, Zeng ZG, Gong HS, He XB, Liu XY, Ma YS, et al. Seasonal variation and sexual difference of home ranges by takins. The Journal of Wildlife Management. 2017; 81(5):938-42.
3) Nowadays, linking activity data (measured by accelerometers) to spatial data (GPS positions) gives new insight into animal behavior (what is an animal doing and at what place?) but in this study, spatial data were used to roughly extrapolate locomotor activity data.

OK. Because of project capital limitation, the Lotak collars GPS7000 of we used were not fitted out with 3D-activity loggers. The previous research on takin activity has had shortcomings concerning the observation period and study method. Fox example, former studie which were carried out by (Li MF et al. 2011 in Sichuan Journal of Zoology) was researched using only 6 camera traps in winter and spring. Therefore, our study first researched locomotion activity pattern of takin thourghout year, seasonal and sexual differrences.
The study has some other significant shortcoming:
1) The GPS recording interval of 2 h (as a minimum) is still very rough for locomotion activity data. The cited study (Ensing et al. 2014) which proves that GPS positions can be used to deduce the activity behavior has been used data measured every hour (which is twice as much data per day like here). Furthermore, Ensing et al. 2014 did include (spatial) error corrections in their calculations; in the manuscript here I did not find any of this.

OK.We accept this. We calculated the true travel distance corrected using average change in altitude between successive locations based on Ensing et al. (2014).
2) In the manuscript, temperature data taken by the collars were defined as the ambient temperature but these temperatures are significantly affected by the heat radiation of the animal wearing the collar, the solar radiation and others influenced. Initially, these temperature sensors were used to determine whether the animal wearing the collar died, causing a drop in the measured temperature and much less daily fluctuations. Thus, a clear measurment of the ambient temperature by the collars is not proven so far. Figure 6 of the manuscript confirms my statement: Monthly mean temperatures measured by the collar (Fig. 6) are quite different from the monthly mean temperatures for January and July given in line 69/70. Before using the temperature data from the collars weared by an animal as ambient temperature data, one should have evaluate their real relationship to the ambient temperatures .

OK.We accept this. We have evaluate their real relationship to the ambient temperatures.
3) In the manuscript, bimodal activity patterns and different seasonal activity levels seem to be caused only by the ambient temperature and the food supply (NDVI) as only these parameters are discussed. Other basic physiological (clock-gene, circadian evolutionary adaptations) and ecological (predators, food competitors) influencing factors are not even mentioned throughout the manuscript.

OK. We accept this. We explained the effect of predators and food competitors for takin.
4) Most analyzes (for instance reference number 34 or the control charts) are quite old-school and - in contrast - badly presented that sometimes I could not fully understand them.

OK. We accept this. We had present the mean daily activity curve (for 24 hours with standard deviation) per season and per sex in which the twilight times was shaded.
5) The measured data are not fully available and - in oposite to the author's statement - also not available in the manuscript. This makes the study incomprehensible for other researchers. For instance, GPS data could be stored in a data base (like Movebank which has a lot of data measured by Lotek collars) from which they are available in some cases also shareable .

OK. We accept this. We had present Takin-specific GPS collar data including individual ID, location ID, date, time, latitude, longitude, height, DOP and temperature from 1 July 2014 to 30 June 2015 in S2_table.
6) English language should be improved, sometimes I couldn´t understand and it was difficult to read.
OK. We accept this.
Additionally some comments to special lines in the manuscript:
line 31: insert a space after "loss"

OK. We accept this.
line 47: studies do not think; maybe replace "think" with "show".

OK. We accept this.
line 70: why general weather data are quoted here from other papers about takins? There are general weather stations whose measurements should serve as a source.

OK. We accept this. We used the temperature data of Foping weather station around the study area from 1981 to 2010.
line 73-79: I miss information about nutrition competitors or predators for the takins.

OK. We accept this. We increased information about nutrition competitors or predators for the takins.
Figure 1: I miss the spatial position of the study area within China or at least longitudes and latitudes. It would be better to show the different individuals (or at least genders) with different colors.

OK. We accept this.
line 88: Were the animals lured into traps beforehand or how was it possible to get to the animals in a shooting distance?

OK. We accept this. The dart rifle using immobilizing anesthetic was used to capture takins at a distance of between 10 and 20 meters while the animals were congregated around a feeding site.
line 90/91 and 96/97: What is body mass of the takins? Please insert their individual body mass in S1 table.

OK. We accept this.
line 91 and line 97: write "body" before "mass"

OK. We accept this.
line 99: "et al." irritates me here.

OK. We deleted “et al.”
line 100: change "in" to "from" (download data FROM the collar)

OK. We accept this.
line 114-121: Which twilight was taken? Once they write about sunrise / sunset (sun stands 0 degrees below the horizon), once about nautical twiligth (6-12 degrees below horizon). Why is a "crepuscular period" explained which does not appear later in the manuscript?

OK. We redefined four period of day: dawn, daytime, dusk and nighttime.
line 150: add an "o" in the first word.

OK. We accept this.
line 152: "to the form" - what does that mean?

OK. We revised these results.
Fig. 2-5: I would prefer to present the mean daily activity curve (for 24 hours with standard deviation) per season and per sex in which the twilight times are either hatched or marked by arrows. So it would be only half as many diagrams (8 pieces) but more information in it (the whole activity patterns over the entire day).

OK. We accept this. We had present the mean daily activity curve (for 24 hours with standard deviation) per season and per sex in which the twilight times was shaded.
line 168-174 & table 1: I do not understand that: In the text (line 168, 170, 173) is spoken by "moving rates", in the table the "effect estimates" are given. Where I can you see the exact formulas, results and significance results of the calculated GLMs? I do not understand this table.

OK. We revised these results.
line 207: delete "for foraging" because you do not know if it was really foraging.

OK. We accept this.

223: Reference 48 is the same as Reference 17

OK. We accept this.
Discussion: Overall, it seems to me that too much is being interpreted into the data. Finally, distances have been measured which the collared animal travelled within 2 hours. There is no information on the behavior of the animal at the measuring points or during the time in between and there are a maximum of 12 measuring points per day. Here, these data are often discussed as if one had measured the foraging behavior but there is no proof for it.

Because takins inhabit steep, complex forested alpine and subalpine areas, it is difficult to explore their activity patterns and time budgets in detail, and therefore there is little published information on the activity patterns of takins. Based on our previous research, takins would mostly forage wihle walking (Zeng et al. 2001. Chinese Journal of Zoology.).
287/288 & 17/18: I'm not quite sure what kind of "efforts" were informed by this study. Please, could you precise this statement!

OK. We deleted this statement.
375: note: "written in Chinese"

OK. We accept this.

412: delete "a" in activity

OK. We accept this.
417: Delete "c" in charts

OK. We accept this.

441-443: Delete it as it is the same reference like numer 17

OK. We accept this.

S1 table: Where are the end dates of collar deployment (like written in the table description)? Add to "r" to "Start" in the ehead of column 4! Would be happy to add the body mass of the individual during collaring. Add "[years]" in the head of column 2. You also have the method of age estimation!
OK. We accept this. Some GPS collars did not work one whole year after 30 June 2015. For keeping data consistent, we used collar data from 1 July 2014 to 30 June 2015. The end dates of collar data was 30 June 2015 (like written in the table description). Therefore, we did not list in the table. We could estimate age of takin based on their degrees of wear.
Reviewer #2: Review
Locomotor activity patterns of takin (Budorcas taxicolor) in a temperate mountain region
This study is about the locomotor activity patterns of takin and the factors shaping those patterns. The authors show a clear sexual dimorphism in movement rates during the seasons and further provide evidence for a connection between takin movement rates and resource availability. Since this study is the first to employ GPS tracking to observe takin movement it provides new and detailed insights into the behavioural adaptations of this species to ecological conditions, intrinsic requirements and life history. Especially since the study species is highly endangered the conservational value of this study should be emphasized.
However, I have some concerns with the manuscript in its current form that need to be addressed:
1) The statistical analysis raises the concern of over fitted generalized linear models which weakens the results and makes me wonder if you can trust them at all. Calculating GLMs with four predictor variables on a sample size of ten is unwise, because the over fitting of the model increases the chances that your regression coefficients represent the noise rather than the genuine relationships in the population. Additionally, over fitting reduces the generalizability outside the original dataset, which is, especially in the context of the conservational value of the study, problematic. Besides the over fitting of the model, the chosen method itself might not be entirely correct since based on the nature of data sampling there is potential for some variation between the individuals that might need to be addressed. Imploring generalised linear mixed models that incorporate for example the differing durations of collar deployment of the animals in their random structure might be a better choice.
I would advise the authors to thoroughly rethink their statistical analysis and revise the manuscript accordingly.
OK. We accept this. We reconstructed models including the factors (sex, season, temperature and NDVI) using the GLMM procedure, and revise the manuscript accordingly.
2) The whole manuscript needs to be revised language wise. Especially parts of the introduction and discussion are hard to follow because language errors distract from the actual text. This is not a major issue and can, in my opinion, be easily fixed. I did comment on a few of the language problems (see below), but would advise the authors to have the manuscript proof read by a native speaker if possible.
OK. We accept this.
I have some minor concerns which are addressed in the following:
Line 9: Movement rates can not show differences, the animals or sexes can show differences in their movement rates.
OK. We accept this.
Line 11-13: This sentence does not make sense and is grammatically incorrect. Please rewrite it.
OK. We accept this.
Line 21-23: I think you mean ”Investigating locomotor activity patterns”. The patterns themselves can not reveal anything, only if they are looked at the and put together with other factors can they reveal information.
OK. We accept this. We revised this paragraph.
Line 55: change “recently” to “recent”
OK. We accept this.
Line 68: change „2,904“ to „2.904“
OK. We accept this.
Line 87-88: Is the unbalance of individuals being collard in different years and the resulting differences in the duration of collaring problematic? Please explain how you approached that.
Although takins were collard in different years, we only used all collar data from 1 July 2014 to 30 June 2015, other collar data was not used in this research.
Line 96-97: Were the collars removed after the tracking period? How many collars were retrieved?
The collars still worked after 30 June 2015, but only six collars worked untill 30 June 2015, other four collars did not work one whohe year 30 June 2015. Therefore, we only used all collar data from 1 July 2014 to 30 June 2015. Finally, we retrieved eight collars, other two lose.
Line 98: delete „was“
OK. We accept this.
Line 99: delete the comma after date and the “et al.”
OK. We accept this.
Line 109: In your supplement table S1 I can not see the end dates of collar deployment for each individual, although your table header says that they should be in there. Does that mean that animals were collared for close to, or in some cases over a whole year?
The takins were collared over one whole year, but four collars did not work one whohe year 30 June 2015. Therefore, we only used all collar data from 1 July 2014 to 30 June 2015.
Lines 137-147: I am concerned with your statistics, the way I understand it you calculated models for each response variable with the same four fixed factors (sex, season, temperature and NDVI), based on your rather small sample size of 10 animals I think your models are over fitted. Based on the common rule of thumb for linear regressions, there should be 10 observations per predictor, which would reduce the number of possible predictors in your models drastically.
Furthermore, since your animals were collared in two different years and therefore had different durations of wearing the collars it might be wise to incorporate that potential source of variation into your models by calculating GLMMs instead of GLMs.
OK. We accept this. We reconstructed models including the factors (sex, season, temperature and NDVI) using the GLMM procedure, and revise the manuscript accordingly.
Line 151: returned to what form?
OK. We revised these results.
Line 171-172: “throughout day and during daytime” is the same thing, you do not need this doubling, or does during day mean during the whole period of 24 hours?
OK. We revised these results.
Line 172: contrary to the movement rates at night-time which were higher for males
OK. We revised these results.
Table 1: Predictor and fixed effects are the same thing, what you are showing in the column “fixed effects” are the factor levels of each predictor/fixed effect.
OK. We revised this table.
Line 183: delete “both”
OK. We accept this.
Line 234: “for cold” does not make sense, I think you mean that they retreat to warmer resting places to avoid being exposed to the cold.
OK. We accept this.
Line 279: change “pregnancy” to “pregnant”
OK. We accept this.
Line 280: infant is a rather anthrophonic terminus, maybe use juvenile, calve or young.

OK. We accept this.
