# Supplementary material for: Locomotor activity patterns of takin (Budorcas taxicolor) in a temperate mountain region
Source: PLoS One. 2020 Jul 13;15(7):e0235464. doi: 10.1371/journal.pone.0235464 (PMC7357786; doi:10.1371/journal.pone.0235464)
Supplement: Attachment — Submitted filename: Response to Reviewers.docx [file pone.0235464.s004.docx]

Reviewer #1: After revision, the manuscript has improved significantly. Nevertheless, not all comments were fully clarified and there are some deficiencies in the study. The authors already published several results of their studies on takins (Yan et al. 2016 & 2017, Zeng et al. 2001, reference no. 19-21, 25, 30, 31, 39, 48, 54), thus this manuscript describes the results on the daily and seasonal pattern of the movement rate of takins. In general, it does not contain any significant new findings, even the authors themselves name 5 published studies on the activity pattern of takin (reference nos. 24-28).
The authors' lack of criticism of their own measurements and analyses is striking: Although the quite considerable (systematic) GPS error has been determined, it receives no further attention in the analysis or discussion. The error of the temperature data was not determined at all (I estimate it to be even higher in percentage than the GPS error). The measurement interval of 2 hours is very rough, but despite all these shortcomings, the authors calculate their movement rates to centimeter accuracy (Table 1) and estimate ecological effects without including calculated measurement errors. That is not state-of-the-art.
I have my doubts whether the statistical investigations are really scientifically correct: NDVI, temperature and season certainly correlate strongly, has this been taken into account? Why sometimes the maximum and sometimes the average movement rates were used, could no uniform value be found? The sample sizes from which the test data were calculated differ in the individual categories: male n = 4, female n = 6, different season durations (line 70-72) and day period durations (day, night, dusk, dawn), different total number of values per individual (from M3 = 2348 data = 53.6% of the maximum possible data in the measurement period, up to F6 = 4051 data = 92.5%). Systematic errors (GPS errors) of considerable size (in relation to the calculated movement data) were not taken into account in the analyses. Therefore, the variance within the data of a category can be much greater, which would definitely have an impact on the statistical results.
Line 7: Delete "would

OK. We accept this.

- Line 110: In which habitat and how exactly this GPS error was recorded? Is this value an average value from several measurements? What are the maximum values of the GPS error and how were outliers (maximum GPS errors) detected or filtered out of the GPS measurement data. Why was this GPS error not considered in the further analyses? On the one hand this GPS error seems to be very small for a typical Takin habitat (dense mountain forest). On the other hand, this error (about 11m) is very high compared to the calculated movement rates (on average about 60m/h or never more than 120m/h), so that an inclusion of this GPS error in the analyses would certainly be relevant for the results.

OK. We accept this. We analyzed the effect of this GPS error for movement rate difference in our results.

- Line 111-119: I think the sampling rate (12 values per day) is too low. The method´s reference study of Ensing et al. 2014 had a double sampling rate (24 values per day) and the authors themselves mention that the sampling rate has a decisive influence on the results (line 116/117). It is not known what the animals really did between the single measurements: a movement rate of zero m/hour could be based by different behaviors of the animal like lying or feeding at the same spot, running in circles, moving within the GPS error distance and so on. The greater this time span between the measurements, the greater the nescience. I already mentioned this in the first review, but the authors ignored this objection.

OK. We accept this. Based on Zeng et al. (2001), takins would feed while walking. When lying at the same spot, takins had a rest or ruminated. Therefore, movement rate would indicate feeding activity of takins.

Because of project capital limitation, the Lotak collars GPS7000 of we used were simple. One collar was scheduled to acquire the position every one hour in August 2013. However, this collar did not work before June 2014. Therefore, the collars of we used in this research were scheduled to acquire the position every two hours in June 2014. In these 10 collars, only 4 collars had worked in June 2016; other collars did not work because of battery running out before June 2016.

- Line 123-126: with this definitions, each dusk or dawn phase takes exactly 1.5 hours which is not in reality. Why was this division made this way? To get at least one GPS-location in each twilight phase? Estimating the effects, I would not have chosen the categories "dusk" and "dawn", because these twilight periods are too short for the sampling rate of 2 hrs. Instead, it would have been more useful to concentrate on the comparison "day" to "night" and hide the measurement around the twilight period. The activity peak in the twilight periods can be seen well in figure 2 anyway, the reason for using the categories "dawn" and "dusk" in the statistical tests was not explained and is not understandable to me.

OK. We accept this. We did not choose the categories “dusk” and “dawn”. We would choose the categories “daytime” and “nighttime”.

- Line 127-129: Were 2 steps (one ends during the twilight time, the other begins during the twilight time) added to the respective twilight in this way? If so, the movement rates calculated from a total of 4 measurement intervals (in a total of 8 hours!) were attributed to the twilight phases; this is much longer than the duration of dusk and dawn in reality. In addition, the sample size per day segment differs: dusk/dawn with n = 1 each, "day" with n = 3-5, "night" with n = 5-7. Did you consider this misbalance in your analyses?

OK. We accept this. We did not choose the categories “dusk” and “dawn”.

- Line 144-146: The data and test results for this correlation calculation are missing. Our own investigations with collar temperature data on animals in an enclosure and 2 temperature measurement stations (one in the shade, one in the sun) in this enclosure have shown that although these data correlate in the course of the day and year, there often occur considerable deviations of >10 degrees from the ambient temperature (e.g. if the outside temperatures exceed or fall below certain extreme values, if the collared animal takes a bath, if it moves and heats up or if the collar heats up in direct sunlight). The temperature collar data are therefore only suitable for rough analyses and comparisons (e.g. of daily averages). For using the collar temperature data on a smaller scale (like in this study), it is essential that a corresponding error interval is specified. This error in the temperature measurements may be quite high - similar to the GPS error in this study - and thus will also be relevant for the results. I guess it is well known that if 2 parameters "correlate significantly positive", this still does not mean that these two parameters are identical! Since in this study the analyses were made with the temperature data of the collars, these should not be called "ambient temperature" data (see line 147-149) just because they correlate with them!

OK. We accept this. The temperature data taken by collars were mostly affected by the solar radiation, shade and other factors of GPS location microenvironment, although it also affected by the heat radiation of the takins wearing the collar. Therefore, we called this temperature as “microenvironment temperature (MET)”.

- Line 152-156: The use of the parameters used in the statistical analyses (maximum or average movement rate) was not justified and is incomprehensible to me.

OK. We accept this. We deleted this section.

- Line 201-204, 252-254: How can there be this negative correlation between temperature and movement rate when the movement rate is always higher during the warm season or day period (summer, daytime) than during the cold ones (winter, night)?

OK. In summer, temperatures are at their highest and takins move upward to forage in high altitude regions. Though temperatures in summer were highest during the year (Fig. 4), the takin movement rate was negatively correlated with MET during daytime (Table 1), indicating that the animals prefer to inhabit a cooler environment. The decrease in activity level in response to MET as a strategy against over-heating has previously been researched in other ungulates. During nighttime in winter, the takin movement rate was also negatively correlated with ambient temperature (Table 1), indicating that takins search for warm night habitats to avoid being exposed to the cold. Maintaining thermal balance is most important for the takins to select suitable habitats in winter.

- Line 234: "crepuscular" and "bimodal" mean the same thing.

OK. We accept this. We deleted “bimodal”.

- Line 224-226 & line 314-316: Isn't the seasonal change closely correlated with the change in temperature and NDVI? In this respect, you have simply noticed seasonally varying rates of movement, right? Since the length of day also correlates with seasonal change, I guess you would also find significant correlations with the movement rate if you used this parameter in the analyses.

OK. We accept this. We deleted these words.

- Line 279: "strategy" is misspelled.

OK. We accept this.

- Discussion (line 237-240, line 256-259, line 271-274...): These statements about the behavior of the animals (midnight activity peak, sunbathing, feeding, rumination) or the exact time of certain activity peaks cannot be proven on the basis of the measured data and are therefore rather speculative. For such statements, either acceleration data (behavioral recording) must be measured and/or the sampling rate must be increased.

OK. We deleted this section of Line 237-240.

The takins prefer to lie down and bask long time in the sun in winter. This sunbathing of takins was often observed in winter based our field observation in many years, although we did not systematic analyzed this behavior. Therefore, we still retained this section.

We revised section of 271-274.

- Table 1: For what reason were these 6 parameters (in the first column) chosen? Which sample sizes are behind the individual values (these are certainly different sizes)? The test results are presented too confusingly.

OK. We accept this. We deleted this section.

- Figure 1: I would mention in the description that there were 6 females and 4 males.

OK. We accept this.

- Figure 2: Is the unit (m/h) correct, because the data itself were collected every 2 hours?

OK. We first calculated the Euclidean distance between successive locations (m). We subsequently calculated the true travel distance corrected using average change in altitude between successive locations. Finally, we calculated movement rate by dividing the true travel distance by the time elapsed between them (h).
- Figure S1: In the manuscript, the description or reference to the method of age estimation of the takins is missing. In the table heading "mass" should be replaced by "body mass"

OK. We accept this.

Reviewer #2: This is a revised version of a manuscript on the locomotor activity patterns of takin. Although it is a smaller study with basic hypotheses, the deployment of GPS tracking, as a first for this species, and the correlation of space use with abiotic factors such as NDVI and temperature give the study value and make it a good contribution to its field.
The authors made a good effort to thoroughly revise the manuscript. Especially the language improved a lot. I can also see an effort to improve the statistical analysis, but still have some major concerns with it:
Although adopting a GLMM approach accounts for variation between individuals using the same model structure as before does not fix your problem with overfitting the model! You still have four factors in a model with a sample size of ten. Furthermore with calculating a GLMM you also add another factor as a random factor, further increasing the overfitting of the model. I do not think that your models are reliable at all. You have to reduce the number of fixed factors in your models. Additionally, there are not enough information on the models you ran (random factor? Distribution family? Link function?).

OK. We accept this. We used one-way ANOVA to compare seasonal differences on movement rates during daytime, nighttime and whole day. We constructed models including the factors (sex, MET and NDVI) using the GLMM procedure in each season, random factor being animal individual.
I have some minor concerns which are listed in the following:
Line 8: Change „difference“ to „differences“

OK. We accept this.

Line 10-11: This sentence is still not correct. It is not a complete sentence. If you say “on the contrary during night time” you can not let the sentence just end, the sentence needs to include also what is different during night time. If you want the half sentence concerned with the night time to express, that during night time the movement rate of females was not significantly higher than that of males you can use “compare”: “In spring, the female movement rate was significantly higher than that of males during daytime compared to the movement rate during nighttime” or “In spring, the female movement rate was significantly higher than that of males during daytime but not during nighttime”
Or if during night time the movement rate of males was higher than that of females use “while”: “In spring, the female movement rate was significantly higher than that of males during daytime while during nighttime the movement rate of males was higher than that of females”
OK. We accept this.

Line 46: Change “active” to activity
OK. We accept this.
Line 68: Was there no weather data available during your study period?
OK. We accept this. We had acquired temperature data of the Foping weather station from 1981 to 2016.

Line 108-109: Please state in the manuscript why you chose this specific time frame for your study.

OK. We accept this.

Line 139: change “Arcgis” to “ArcGIS”

OK. We accept this.

Line 161-162: Die you statistically analyze whether there are seasonal differences in the time interval between movement peaks? If not you can not make a statement about whether or not it was longer during spring and summer compared to winter.

OK. We accept this. We deleted this section.

Line 177: Did you do post hoc testing to identify differences between groups? It is not clear were the differences between groups come from since the Friedman test should only provide a result for whether or not there is a difference between groups.

OK. We accept this. We deleted this section.

Line 205: please add a “the” before day

OK. We accept this.

Line 206-207: It is enough to simply state “…was not correlated with ambient temperature.”

OK. We accept this.

Line 230-231: You already stated in line 227 that you found a crepuscular activity pattern, you do not have to say it here again.

OK. We accept this. We deleted this section.

Line 287: change “significantly” to “significant”

OK. We accept this.
Line 298: locomotor ability? Locomotor activity? Locomotor is an adjective and needs a noun in the sentence.

OK. We accept this.
Line 304-305: You stated above that females mainly give birth and lactate during spring, here you are contradicting your previous statement. Which is it? Or do some females give birth in winter and others in spring?

OK. The birth season of takins occurred from February to April. Mother-infant relationship was very close when calf was infantile. In spring, calf moved with their mother. Mother gave care to her infant.
